# Effect of high-quality nursing on alleviating depression and anxiety in patients with thyroid cancer during perioperative period: A protocol for systematic review

**DOI:** 10.1097/MD.0000000000023018

**Published:** 2020-11-06

**Authors:** Ying Cui, Yi-xuan Li

**Affiliations:** a Thyroid Surgery Care Platform, The First Hospital of Jilin University; b Department of Outpatient, The First Hospital of Jilin University, Changchun, China.

**Keywords:** anxiety, depression, nursing, thyroid tumor

## Abstract

**Background:**

This study will assess the effect of high-quality nursing (HQN) on alleviating depression and anxiety (DA) in patients with thyroid cancer (TC) during perioperative period (PPP).

**Methods:**

We will search the following electronic databases (MEDLINE, EMBASE, PsycINFO, Cochrane Library, CINAHL, Web of Science, CBM, WANGFANG, and CNKI) from inception to the present and other literature sources without language limitation. All potential randomized controlled trials reporting on effect of HQN on DA in patients with TC during PPP will be considered for inclusion. Two researchers will separately carry out study selection, data extraction, and study quality evaluation. Any different opinion will be solved by a third author through discussion. All statistical analysis will be performed by RevMan 5.3 software.

**Results:**

We will appraise the effect of HQN on DA in patients with TC during PPP through assessing outcomes of depression, anxiety, pressure, quality of life, and adverse events.

**Conclusion:**

This study will provide evidence to determine whether HQN is effective or not on DA in patients with TC during PPP.

**OSF registration:**

osf.io/sb5r8.

## 1 Introduction

Thyroid cancer (TC) is a very common endocrine malignancy,^[1–3]^ which is characterized by different biological and clinical features.^[4–6]^ It is also the leading cause of mortality and morbidity around the world.^[7–8]^ It consists of 4 subtypes of papillary thyroid cancer, follicular thyroid cancer, medullary thyroid cancer, and anaplastic thyroid cancer.^[9–11]^ It accounts for 3.4% of all diagnosed cancers globally each year.^[12]^ Treatment of TC comprises of medication, radiotherapy, chemotherapy, and surgery.^[13–18]^ Many patients are report to experience depression and anxiety (DA) during the perioperative period (PPP).^[19–23]^ Luckily, studies suggested that HQN can help relieve this disorder.^[24–26]^ However, no systematic review has explored this issue. Thus, this study firstly evaluates the effect of HQN on DA in patients with TC during PPP.

## 2 Methods

### 2.1 Study registration

We have registered this study on OSF (osf.io/sb5r8). It is reported according to the Preferred Reporting Items for Systematic Review and Meta-Analysis Protocols standards.^[27]^

### 2.2 Eligibility criteria

#### 2.2.1 Type of studies

This study will include randomized controlled trials (RCTs) of HQN on DA in patients with TC during PPP. We will exclude animal study, review, comment, case report, case series, observational study, uncontrolled study, and quasi-RCTs.

#### 2.2.2 Type of participants

We will include patients (over 18 years old) with TC who were diagnosed as depression and/or anxiety without restrictions of race and sex.

#### 2.2.3 Type of interventions and controls

In the experimental group, HQN as the intervention was used for the management of DA in patients with TC during PPP.

In the control group, any management could be used as comparator, such as conventional treatment, or placebo. However, we will exclude combined therapy with any forms of HQN.

#### 2.2.4 Type of outcomes

The primary outcomes include depression (e.g.,Hamilton Depression Scale) and anxiety (e.g.,Hamilton Anxiety Scale).

The secondary outcomes comprise of pressure, quality of life, and any adverse events.

### 2.3 Search strategy

Two researchers will search the electronic databases (MEDLINE, EMBASE, PsycINFO, Cochrane Library, CINAHL, Web of Science, CBM, WANGFANG, and CNKI) from inception to the present without language and publication status restrictions. All potential RCTs examining the effect of HQN on DA in patients with TC during PPP will be included. The detailed retrieval strategy of MEDLINE is built in a Table 1. We will modify this retrieval strategy to other electronic databases.

**Table 1:** Search strategy for MEDLINE.

Number	Search terms
1	thyroid
2	papillary
3	follicular
4	medullary
5	anaplastic
6	cancer
7	carcinoma
8	tumor
9	depression
10	anxiety
11	psychological disorder
12	Or 1-11
13	surgery
14	operation
15	nursing
16	care
17	high quality
18	advanced
19	Or 13-18
20	randomized
21	randomly
22	random
23	controlled trial
24	clinical trial
25	control
26	concealment
27	blind
28	allocation
29	Or 20-28
30	12 and 19 and 29

Additionally, we will also retrieve other literature sources, such as conference proceedings, bibliography lists of associated review, and ongoing trials from website of clinical trial registration.

### 2.4 Data selection and extraction

#### 2.4.1 Study selection

Two researchers will independently carry out study selection. First, titles/abstracts of all searched studies will be screened to remove duplicated and irrelevant studies. Then, full text of all potential articles will be further identified against all inclusion criteria. If there are divergences between 2 researchers, we will invite a third researcher to solve them. The whole process of study selection will be exerted in a flow chart.

#### 2.4.2 Data extraction

Two researchers will independently extract data using a data collection template. We will extract data of study information (title, first author, time of publication, location, et al), study characteristics (study design, study setting, sample size, study methods, et al), patient characteristics (race, sex, age, tumor types, inclusion and exclusion criteria, et al), interventions and controls (treatment types, dosage, duration, route of administration, et al), outcomes (primary and secondary outcome indicators, dropouts, follow-up information, et al), and conflict of interest. We will invite a third researcher to resolve any division. If any unclear or missing data occurs, we will contact primary authors to obtain it by email.

### 2.5 Assessment of risk of bias

The methodological quality of all included study will be appraised by 2 independent researchers according to the Cochrane Risk of Bias Tool. It covers 7 domains, and each one is judged as low, unclear or high risk of bias. Any divergence will be solved by a third researcher through discussion or consultation.

### 2.6 Statistical analysis

This study will utilize RevMan 5.3 software for statistical analysis. We will analyze outcome results of continuous data as mean difference (MD) or standardized MD and 95% confidence intervals (CIs), and of dichotomous data as risk ratio and 95% CIs. All statistical heterogeneity will be assessed using *I*^*2*^ test. It is explained as follows: *I*^*2*^ ≤ 50% exerts reasonable heterogeneity, and we will select a fixed-effects model; *I*^*2*^ > 50% indicates substantial heterogeneity, and we will choose a random-effects model. If reasonable heterogeneity is identified and sufficient data are collected from included studies, meta-analysis will be performed. On the other hand, if significant heterogeneity is found, we will perform subgroup analysis to explore its possible reasons. If there is still substantial heterogeneity after subgroup analysis, we will not pool the data. Instead, we will conduct a narrative analysis given the presence of significant heterogeneity.

### 2.7 Subgroup analysis

We will employ a subgroup analysis based on the different types of tumor, variations in study and patient characteristics, and differences in treatments and controls.

### 2.8 Sensitivity analysis

We will undertake sensitivity analysis to test the robustness and stability of merged outcome data by omitting studies with high risk of bias.

### 2.9 Reporting bias

If over 10 trials reporting the same outcome, we will carry out a funnel plot to examine potential reporting bias,^[28]^ and will perform Eggers regression test^[29]^ to check funnel plot asymmetry.

### 2.10 Ethics and dissemination

No ethical approval is required for this study, because it will not collect individual patient data. We will publish this study on a peer-reviewed journal.

## 3 Discussion

Previous studies suggested that HQN can help relieve DA in patients with TC during PPP. With the increasing number of evidence supporting its effect on DA in TC during PPP, there is a need to pool evidence from eligible studies to accurately assess the treatment effect. This is the first comprehensive and systematic study to incorporate different outcome data into analyses. We will search as comprehensive as possible trials in both electronic databases and other literature sources to avoid losing potential studies. The results of this study may provide evidence to support the effect of HQN on DA in TC during PPP. However, this study may still have several limitations. First, the sample size of eligible trials may be small. Second, the quality of included studies may be poor. Third, the statistical heterogeneity across trials may be too high to synthesize the data. All those restrictions may affect the findings of this study.

## Author contributions

**Conceptualization:** Ying Cui, Yi-xuan Li.

**Data curation:** Ying Cui, Yi-xuan Li.

**Formal analysis:** Ying Cui, Yi-xuan Li.

**Investigation:** Ying Cui.

**Methodology:** Yi-xuan Li.

**Project administration:** Ying Cui.

**Resources:** Yi-xuan Li.

**Software:** Yi-xuan Li.

**Supervision:** Ying Cui.

**Validation:** Ying Cui, Yi-xuan Li.

**Visualization:** Ying Cui, Yi-xuan Li.

**Writing – original draft:** Ying Cui, Yi-xuan Li.

**Writing – review & editing:** Ying Cui, Yi-xuan Li.

## References

[1] Cabanillas ME McFadden DG Durante C Thyroid cancer. Lancet 2016;388:2783–95. 27240885 10.1016/S0140-6736(16)30172-6

[2] Carling T Udelsman R Thyroid cancer. Annu Rev Med 2014;65:125–37. 24274180 10.1146/annurev-med-061512-105739

[3] Lebastchi AH Callender GG Thyroid cancer. Curr Probl Cancer 2014;38:48–74. 24951026 10.1016/j.currproblcancer.2014.04.001

[4] Berdelou A Lamartina L Klain M Treatment of refractory thyroid cancer. Endocr Relat Cancer 2018;25:R209–23. 29371330 10.1530/ERC-17-0542

[5] Schlumberger M Thyroid cancer. Rev Prat 2017;67:661–2. 30512741

[6] Sessions RB Davidson BJ Thyroid cancer. Med Clin North Am 1993;77:517–38. 8492607 10.1016/s0025-7125(16)30237-1

[7] Nagayama Y Thyroid cancer. Nihon Rinsho 2012;70:436–40. 22514922

[8] Hill C Schlumberger M Epidemiology of thyroid cancer. Rev Prat 2017;67:668–72. 30512743

[9] Thorneycroft IH Thyroid cancer. Clin Obstet Gynecol 2002;45:879–83. 12370629 10.1097/00003081-200209000-00034

[10] Clark OH Duh QY Thyroid cancer. Med Clin North Am 1991;75:211–34. 1987444 10.1016/s0025-7125(16)30480-1

[11] Burns WR Zeiger MA Differentiated thyroid cancer. Semin Oncol 2010;37:557–66. 21167375 10.1053/j.seminoncol.2010.10.008

[12] Chmielik E Rusinek D Oczko-Wojciechowska M Heterogeneity of Thyroid Cancer. Pathobiology 2018;85:117–29. 29408820 10.1159/000486422

[13] Elisei R Schlumberger MJ Müller SP Cabozantinib in progressive medullary thyroid cancer. J Clin Oncol 2013;31:3639–46. 24002501 10.1200/JCO.2012.48.4659 PMC4164813

[14] Tahara M Kiyota N Yamazaki T Lenvatinib for Anaplastic Thyroid Cancer. Front Oncol 2017;7:25. 28299283 10.3389/fonc.2017.00025 PMC5331066

[15] Sherman EJ Lim SH Ho AL Concurrent doxorubicin and radiotherapy for anaplastic thyroid cancer: a critical re-evaluation including uniform pathologic review. Radiother Oncol 2011;101:425–30. 21981877 10.1016/j.radonc.2011.09.004

[16] Advani SH Hege UP Leukemia after 131I treatment of thyroid cancer--comments on the article 'Second cancer following chemotherapy and radiotherapy--an epidemiological perspective’ by J. Kaldor. Acta Oncol 1992;31:65. 1586507 10.3109/02841869209088269

[17] Iyer NG Shaha AR Management of thyroid nodules and surgery for differentiated thyroid cancer. Clin Oncol (R Coll Radiol) 2010;22:405–12. 20381323 10.1016/j.clon.2010.03.009 PMC4806860

[18] Tennvall J Lundell G Hallquist A Combined doxorubicin, hyperfractionated radiotherapy, and surgery in anaplastic thyroid carcinoma. Report on two protocols. Swedish Anaplastic Thyroid Cancer Group Cancer 1994;74:1348–54. 8055459 10.1002/1097-0142(19940815)74:4<1348::aid-cncr2820740427>3.0.co;2-d

[19] Tang H Zhang Y Identification and bioinformatics analysis of overlapping differentially expressed genes in depression, papillary thyroid cancer and uterine fibroids. Exp Ther Med 2018;15:4810–6. 29805500 10.3892/etm.2018.6023 PMC5952074

[20] Badihian S Jalalpour P Mirdamadi M Quality of Life. Anxiety and Depression in Patients with Differentiated Thyroid Cancer under Short Term Hypothyroidism Induced by Levothyroxine Withdrawal Klin Onkol 2016;29:439–44. 27951721

[21] Tagay S Herpertz S Langkafel M Health-related Quality of Life, depression and anxiety in thyroid cancer patients. Qual Life Res 2006;15:695–703. 16688502 10.1007/s11136-005-3689-7

[22] Tagay S Senf W Schöpper N Protective factors for anxiety and depression in thyroid cancer patients. Z Psychosom Med Psychother 2007;53:62–74. 17311732 10.13109/zptm.2007.53.1.62

[23] Tagay S Herpertz S Langkafel M Health-related quality of life, anxiety and depression in thyroid cancer patients under short-term hypothyroidism and TSH-suppressive levothyroxine treatment. Eur J Endocrinol 2005;153:755–63. 16322380 10.1530/eje.1.02047

[24] Wang S Huang H Wang L A psychological nursing intervention for patients with thyroid cancer on psychological distress and quality of life: a randomized clinical trial. J Nerv Ment Dis 2020;208:533–9. 32187128 10.1097/NMD.0000000000001157

[25] Hang JY Wang ZX The current situation and influencing factors of self efficacy in patients with differentiated thyroid cancer treated with radioactive iodine. J Clin Otorhi Head Neck Surg 2017;31:333–7. 10.13201/j.issn.1001-1781.2017.05.002 29871256

[26] Wu HX Zhong H Xu YD Psychological and behavioral intervention improves the quality of life and mental health of patients suffering from differentiated thyroid cancer treated with postoperative radioactive iodine-131. Neuropsychiatr Dis Treat 2016;12:1055–60. 27194911 10.2147/NDT.S105460 PMC4859420

[27] Shamseer L Moher D Clarke M Preferred reporting items for systematic review and meta-analysis protocols (PRISMA-P) 2015: elaboration and explanation. BMJ 2015;350:g7647. 25555855 10.1136/bmj.g7647

[28] Sutton AJ Duval SJ Tweedie RL Empirical assessment of effect of publication bias on meta-analyses. BMJ 2000;320:1574–7. 10845965 10.1136/bmj.320.7249.1574 PMC27401

[29] Egger M Davey Smith G Schneider M Bias in meta-analysis detected by a simple, graphical test. BMJ 1997;315:629–34. 9310563 10.1136/bmj.315.7109.629 PMC2127453

